# Neurovascular Coupling under Chronic Stress Is Modified by Altered GABAergic Interneuron Activity

**DOI:** 10.1523/JNEUROSCI.1357-19.2019

**Published:** 2019-12-11

**Authors:** Kayoung Han, Jiwoong Min, Myunghee Lee, Bok-Man Kang, Taeyoung Park, Junghyun Hahn, Jaeseung Yei, Juheon Lee, Junsung Woo, C. Justin Lee, Seong-Gi Kim, Minah Suh

**Affiliations:** ^1^Center for Neuroscience Imaging Research, Institute for Basic Science, Suwon 16419, Korea,; ^2^Department of Health Sciences and Technology, SAIHST, Sungkyunkwan University, Seoul 06351, Korea,; ^3^Department of Biomedical Engineering, Sungkyunkwan University, Suwon 16419, Korea,; ^4^Biomedical Institute for Convergence at SKKU, Sungkyunkwan University, Suwon 16419, Korea,; ^5^Center for Glia-Neuron Interaction, Korea Institute of Science and Technology, Seoul 02792, Korea,; ^6^Department of Neuroscience, Division of Bio-medical Science & Technology, Korea Institute of Science and Technology School, Korea University of Science and Technology, Seoul 02792, Korea, and; ^7^Center for Cognition and Sociality, Institute for Basic Science, Daejeon 34126, Korea

**Keywords:** cerebral blood flow, chronic stress, GABAergic interneuron, neurovascular coupling, nNOS

## Abstract

Neurovascular coupling (NVC), the interaction between neural activity and vascular response, ensures normal brain function by maintaining brain homeostasis. We previously reported altered cerebrovascular responses during functional hyperemia in chronically stressed animals. However, the underlying neuronal-level changes associated with those hemodynamic changes remained unclear. Here, using *in vivo* and *ex vivo* experiments, we investigate the neuronal origins of altered NVC dynamics under chronic stress conditions in adult male mice. Stimulus-evoked hemodynamic and neural responses, especially beta and gamma-band local field potential activity, were significantly lower in chronically stressed animals, and the NVC relationship, itself, had changed. Further, using acute brain slices, we discovered that the underlying cause of this change was dysfunction of neuronal nitric oxide synthase (nNOS)-mediated vascular responses. Using FISH to check the mRNA expression of several GABAergic subtypes, we confirmed that only nNOS mRNA was significantly decreased in chronically stressed mice. Ultimately, chronic stress impairs NVC by diminishing nNOS-mediated vasodilation responses to local neural activity. Overall, these findings provide useful information in understanding NVC dynamics in the healthy brain. More importantly, this study reveals that impaired nNOS-mediated NVC function may be a contributory factor in the progression of stress-related diseases.

**SIGNIFICANCE STATEMENT** The correlation between neuronal activity and cerebral vascular dynamics is defined as neurovascular coupling (NVC), which plays an important role for meeting the metabolic demands of the brain. However, the impact of chronic stress, which is a contributory factor of many cerebrovascular diseases, on NVC is poorly understood. We therefore investigated the effects of chronic stress on impaired neurovascular response to sensory stimulation and their underlying mechanisms. Multimodal approaches, from *in vivo* hemodynamic imaging and electrophysiology to *ex vivo* vascular imaging with pharmacological treatment, patch-clamp recording, FISH, and immunohistochemistry revealed that chronic stress-induced dysfunction of nNOS-expressing interneurons contributes to NVC impairment. These findings will provide useful information to understand the role of nNOS interneurons in NVC in normal and pathological conditions.

## Introduction

Chronic stress is a known causal factor in the progression of major depressive disorder (Dolan et al., 1994; Ota et al., 2014). Physical and psychological stressors affect the brain system, particularly through activation of the hypothalamus-pituitary-adrenal (HPA) axis, which results in glucocorticoid production (McEwen et al., 2015). Repeated exposure to glucocorticoids affects dendritic remodeling and synaptic changes (Seib and Wellman, 2003; Liston et al., 2006; Shansky et al., 2009). These neural alteration affects neuronal networks and causes functional alterations, such as sensory perception (Khasar et al., 2008; Zheng et al., 2015) and sensory gating circuit in chronically stressed animals (Stevens et al., 2001; Maxwell et al., 2006). One of the chronic stress-associated neuronal alterations that is widely studied is GABAergic interneuron dysfunction. In the mPFC and hippocampus, both the number of GABAergic interneurons and their sIPSCs were reduced after exposure to chronic stress (Hu et al., 2010; Holm et al., 2011; Gilabert-Juan et al., 2013). Since repeated stress exposure also influences the cardiovascular system (Low et al., 2009), recent studies have focused not only on changes in the neural network system but also on alterations in neurovascular coupling (NVC) under chronically stressed conditions. In one instance, the malfunction of inwardly rectifying potassium (K_ir_) channels in parenchymal arteriolar myocytes was observed in chronically stressed mice (Longden et al., 2014). Moreover, our previous work with chronically stressed rats revealed reduced cerebrovascular response following sensory stimulation (Lee et al., 2015).

NVC, the tightly linked relationship between local neural activity and subsequent changes in cerebral blood flow (CBF), is an important function for maintenance of brain homeostasis. NVC enables the microvascular system to meet the metabolic demands created by neural activity (Hillman, 2014). Excitatory neurons, inhibitory neurons, glia, and cerebral vasculature are all components of the NVC unit. Elaborate interplay within these units regulate adequate blood and nutrient supply to localized brain regions (Iadecola, 2017). For example, following the activation of NMDA receptors, pyramidal cells cause an increase in CBF via the COX-2-mediated prostaglandin E2 (PgE2) pathway (Lacroix et al., 2015). In addition to pyramidal cells, GABAergic interneurons have direct contact with nearby parenchymal arterioles. GABAergic interneurons regulate nearby arteriolar diameter through their expression of vasoactive mediators, such as neuropeptide Y (NPY), somatostatin (SOM), and/or neuronal nitric oxide synthase (nNOS) (Cauli et al., 2004; Kocharyan et al., 2008). Optogenetic activation of GABAergic interneurons can provoke an increase in CBF (Uhlirova et al., 2016; Vazquez et al., 2018). These studies imply that any abnormal interactions between GABAergic interneuron activity and vascular functions under pathological conditions may alter hemodynamic signals. Thus, we can speculate that altered GABAergic neuronal function in a chronically stressed brain will affect both overall hemodynamics and NVC.

Previously, we reported that chronically stressed rats exhibit a decrease in cerebral blood volume (CBV) response to a sensory stimulus (Lee et al., 2015). From these observations, we hypothesized that there would be a neuronal activity-originated source that would induce such changes on NVC and modulate vascular tone and function. To explore this hypothesis, we chose primary somatosensory cortex to adopt a validated experimental design from a wide range of NVC imaging research (Berwick et al., 2008; Iordanova et al., 2015). In the present study, we investigate the underlying mechanisms of chronic stress-induced alterations in the NVC relationship. Here, we propose that altered GABAergic interneuron activity, specifically in nNOS-positive interneurons, is the key component in altered neurovascular function observed after chronic stress exposure.

## Materials and Methods

### 

#### 

##### Experimental design.

All experiments were done with C57BL/6N male mice. A total of 240 mice were included in the study: 20 mice (10 control and 10 stressed mice) for *in vivo* OIS imaging and local field potential (LFP) recording experiments, 6 mice (3 control and 3 stressed mice) for blood pressure measurements, 22 mice (12 control and 10 stressed mice) for FISH to measure mRNA expression of each subtype of GABAergic interneuron, 23 mice (12 control and 11 stressed mice) for IHC to measure nNOS and GAD67 protein expression, 33 mice (59 cells from 15 control mice and 68 cells from 18 stressed mice) for patch-clamp recording, and 136 mice (125 slices from 65 control mice and 116 slices from 71 stressed mice) for vascular imaging in *ex vivo* preparation.

##### Animals.

Eight-week-old healthy male C57BL/6N mice (OrientBio) were used. Mice were raised in a cage with *ad libitum* access to food and water. The environment was maintained with a 12 h light/dark cycle (light on 9:00 A.M), 24°C-25°C temperature, and 50%–60% humidity. All experimental procedures were approved by the Institutional Animal Care and the Use Committee of Sungkyunkwan University.

##### Mouse model of chronic restraint stress.

Chronically stressed mouse models were established via the application of chronic restraint stress. This well-established protocol is known to induce depressive-like behaviors (Buynitsky and Mostofsky, 2009). Three weeks of restraint stress was applied to 8-week-old C57BL/6 mice. The mice were immobilized with plastic bags (Decapicones, Braintree Scientific) in their home cages for 6 h per day, starting at 10:00 A.M. During the 6 h restraint period, animals were restricted from food and water intake. The control group mice were allowed to move freely in their individual cages. Each animal's weight and food intake were checked every week.

##### Elevated plus maze (EPM) test.

For behavioral phenotyping, we performed an EPM test at a day after the 3 week restraint stress protocol. The EPM test is often used to measure anxiety-like behavior in stress models (Carobrez and Bertoglio, 2005). The plus maze consisted of four arms (30 cm × 5 cm): two opposite arms were enclosed by 20 cm walls (closed platform) and the other two arms were not enclosed by walls (open platform). Animal movement on the platforms was recorded for 5 min with a video recording and analyzing system (Ethovision XT, Noldus). The time each mouse spent in the open and closed arms was calculated automatically with behavior analysis software. For this experimental study, stress-resilient mice were excluded.

##### Blood sampling and ELISA.

After the end of 3-weeks of stress induction, plasma was collected without stress exposure on the day of collection. Mice (control, *n* = 25; stress, *n* = 25) were briefly anesthetized with 3% isoflurane (Hana Pharm) using an anesthesia machine (VetEquip), and trunk blood was collected in heparin-coated tubes (BD Vacutainer, Becton Dickinson). Blood samples were centrifuged at 5000 rpm for 10 min. The concentration of corticosterone in the plasma was analyzed using a corticosterone ELISA kit (Assaypro). Assay procedures were followed according to the instructions provided in the kit. The absorbance at a wavelength of 450 nm was scanned by a microplate reader (Synergy HT, BioTek). A standard curve was generated using standard solutions, and the sample concentrations were determined from the standard curve.

##### Blood pressure measurement.

Blood pressure was measured using a tail-cuff blood pressure measurement system (Kent Scientific). Anesthesia was induced with 3% isoflurane and maintained with 1.3 mg/kg urethane (U2500, Sigma-Aldrich) to achieve a state similar to optical intrinsic signal (OIS) imaging and LFP recording experiments. Body temperature was maintained at 37°C using a homeothermic heating pad system (FHC). Systolic, diastolic, and mean blood pressure of individuals were calculated as an average of 30-cycle measurements.

##### Animal surgery for *in vivo* experiments.

For simultaneous measurement of hemoglobin-based OIS and neural activity, we performed a 3-mm-diameter craniotomy on an area over a region corresponding to forelimb response in the right primary somatosensory cortex (right S1FL). Animals were anesthetized with isoflurane using an anesthesia machine (VetEquip); 3% isoflurane was used for induction, and the isoflurane level was maintained at 1.5% during the surgery. For head fixation, a customized chamber frame was mounted on the skull. A dental resin wall was built around the site of craniotomy. Body temperature was maintained at 37°C-38°C using a homeothermic heating pad system (FHC). After the surgery, the mice were anesthetized with urethane (1.3–1.4 mg/kg, i.p.) during OIS imaging and electrophysiology experiments. The use of anesthesia was intended to avoid acute stress effects on neurovascular responses caused by head fixation during CBV-weighted optical imaging and electrophysiological recordings. Urethane is known to induce a highly stable state of anesthesia (Boorman et al., 2015). It also preserves both excitatory and inhibitory synaptic transmission (Sceniak and MacIver, 2006) and NVC (Berwick et al., 2008) with relatively limited effect on the cardiovascular system (Janssen et al., 2004). Throughout experiments, we monitored animal anesthesia level with the toe pinching test and indicators of physiological status (PhysioSuite, Kent Scientific).

##### OIS imaging.

Hemodynamic response to electrical forelimb stimulation was recorded with an optical imaging system (Imager 3001-Celox, Optical Imaging). The exposed cortex was filled with silicone oil (Sigma-Aldrich) and illuminated with an LED lamp (CLS150, Leica Microsystems). Images were collected with a 10 Hz frame rate using a fast acquisition camera (Photonfocus AG) through 50 mm tandem lenses. Since 546 nm is the isosbestic wavelength in the absorption spectra of oxyhemoglobin and deoxyhemoglobin, reflected light was filtered with a 546 ± 30 nm bandpass filter to measure the total amount of hemoglobin (Hbt) (Horecker, 1943). The pixel resolution of the field of view was 372 × 372 pixels, and the pixel size was 8 μm.

For electrical forelimb stimulation, two custom needle electrodes were placed in the left forelimb between digits 2 and 4. The stimulus was applied for 20 s using a stimulation generator (Master-9, A.M.P.I.) and consisted of 500 μs electrical pulses at 4 Hz frequency with 0.5 mA amplitude. The activated area within the right S1FL region was identified; then, the insertion site of the electrode for electrophysiology was decided. The intertrial interval was 100 s, and the trials were repeated for 18 times per animal (control, *n* = 10; 3 week stress model, *n* = 10).

Intensity changes were computed for each trial with the baseline defined as the 5 s period preceding the stimulus onset. The ROI was set as the 5 × 5 pixels nearest to the position of the electrode tip avoiding surface vessels. Time series data were temporally averaged to each time point. The peak amplitude was determined within the stimulation period. The onset time was determined to be the time point at which the intensity change exceeded the baseline average by 2 SDs. The summated image of 18 trials, representing 1 animal, was binned by 4 × 4 pixels. Pixel counts within the top 50% of the value of intensity change were used to calculate the spatial extent of the active area.

##### LFP recording.

An electrode with ∼0.5 mΩ impedance (FHC) was placed within the activation area of the right S1FL region at a depth of 300 μm, targeting layer II/III of the somatosensory cortex. Electrophysiological activity during forelimb stimulation was recorded at 40 kHz frequency (Plexon). The raw electrophysiology data were filtered between 0.5 and 200 Hz for LFP. The amplitudes of the LFPs induced by each electrical pulse were summed >80 electrical pulses during a 20 s period of stimulation, then averaged for repeated trials. LFP analyses were performed by using the open-source Chronux 2.12 toolbox. LFP spectra were calculated by using five tapers and 1 s time windows with a step size of 50 ms. The frequency ranging from 2 to 4 Hz was taken to be the delta band, 4 to 7 Hz to be the theta band, 7 to 13 Hz to be the alpha band, 13 to 30 Hz to be the beta band, and 30 to 100 Hz to be the gamma band. The frequency ranging from 55 to 65 Hz was excluded from all analyses due to a notch filter at 60 Hz.

##### Brain slice preparation.

Mice were decapitated after isoflurane anesthesia. The brains were quickly removed and placed into ice-cold ACSF containing 93 mm NMDG, 2.5 mm KCl, 1.2 mm NaH_2_PO_4_, 30 mm NaHCO_3_, 20 mm HEPES, 25 mm glucose, 5 mm sodium ascorbate, 2 mm thiourea, 3 mm sodium pyruvate, 10 mm MgSO_4_, and 0.5 mm CaCl_2_, adjusted to 300–310 mOsm, pH 7.4, and continuously bubbled with 95% O_2_/5% CO_2_. Coronal slices (300 μm thick) containing the somatosensory cortex were prepared using a vibratome (VT 1200S, Leica Microsystems). Slices were then incubated at room temperature for at least 1 h in oxygenated ACSF containing 124 mm NaCl, 2.5 mm KCl, 1 mm NaH_2_PO_4_, 24 mm NaHCO_3_, 10 mm glucose, 1 mm sodium ascorbate, 2 mm sodium pyruvate, 2 mm MgSO_4_, and 2 mm CaCl_2_. Slices were then transferred to a submerged recording chamber circulated with oxygenated ACSF using a peristaltic pump (Ismatec). All *ex vivo* experiments were conducted under the condition of 95% O_2_. It is noted that, although the condition of 20%–30% O_2_ is recommended for young mice (Gordon et al., 2008; Hall et al., 2014; Mehina et al., 2017), our preliminary studies showed that our adult brain slices under a condition of 20%–30% O_2_ did not well exhibit preconstriction of penetrating arterioles by 9,11-dideoxy-9a,11a-methanoepoxyprostaglandin F_2α_ (U46619). Hence, the protocol using 95% O_2_ was chosen for our adult brain slice studies.

##### Vessel imaging in brain slices.

Penetrating arterioles (8–14 μm in luminal diameter) in layer II/III of the somatosensory cortex were selected under infrared illumination, using a 63 × 0.9 NA water objective lens (Leica Microsystems). Vascular dynamic images were acquired every second on a microscope (DM6000 FS, Leica Microsystems) with a digital CMOS camera (ORCA-Flash4.0, Hamamatsu) at room temperature. The pixel resolution of the FOV was 2048 × 2048 pixels with pixel size measuring 0.08 μm. Since acute brain slices have no vascular tonic movement, blood vessels were preconstricted by circulating a thromboxane A2 receptor agonist, U46619 (65–100 nm, Cayman Chemical). As the degree of vasoconstriction has been reported to influence the amplitude and polarity of vascular responses, only arterioles developing a similar and stable vasoconstriction (25%–30% constriction level) within the 20 min following U46619 application were included in the analysis. Electrical stimulation used a stimulation generator (Master-9, A.M.P.I.) and consisted of 300 μs electrical pulses at 20 Hz frequency with 2 V amplitude.

##### Vascular reactivity analysis in brain slices.

Before the quantification of vascular luminal diameter changes, we used ImageJ (RRID:SCR_002285) for image rotation, Gaussian filtering, registration, and generating SD maps. Specifically, all stack images were rotated to align vascular orientation vertically. Then, to remove red blood cell signal present in the vascular lumen, we applied a temporal Gaussian filter with a 9-slice FWHM kernel. After correcting for the red blood cell signal, all images were realigned using the “StackReg” plug-in in Fiji to compensate for potential in-plane movements. Finally, using the filtered and realigned image, we generated a temporal SD map to find a stably changing luminal area during stimulation.

Next, the changes of luminal diameter were quantified from a registered image using a custom analysis method developed in MATLAB (The MathWorks, RRID:SCR_001622). First, we selected three lines perpendicular to the vascular lumen. The perpendicular line selection was determined under two conditions: (1) the line should be located at the stably changing luminal regions as determined by the SD map; and (2) the distance between each line should be at least 10 μm to avoid selecting vascular lumen controlled by identical smooth muscles. For each selected line, an average line was recalculated by averaging the values within the area 0.4 μm above and below each line. A 2D line stack image was created by evolving the averaged line across all frames. To sum up, the three lines perpendicular to the vascular luminal area are arranged in temporal order to create three line-stack images for every vascular image stack.

The generated 2D line stack image exhibits vascular luminal boundaries on both sides along the temporal axis. Therefore, calculating the distance between the two defined luminal boundaries allows for measurement of the diameter change of vascular lumen over time. To find the coordinates of the luminal boundaries, we performed an “edge contrast enhancement” for clarified boundary contrast. Then, we found two coordinates which have “local minima” near each luminal boundary. In this way, we defined the coordinates at all temporal axes and quantified the distance between the luminal boundaries over time. We then converted the quantified diameter change into percentage change using the initial baseline of the first minute.

##### Whole-cell recording.

Whole-cell recordings of pyramidal neurons in the somatosensory cortex were acquired in acute coronal brain slices. Borosilicate glass pipettes (BF100–58-10, Sutter Instrument) with resistances ranging from 5 to 8 mΩ were pulled using a laser micropipette puller (P-1000, Sutter Instrument). For sEPSCs, the pipette was filled with an internal solution containing 135 mm K-gluconate, 4 mm KCl, 2 mm NaCl, 10 mm HEPES, 4 mm EGTA, 4 mm Mg-ATP, 0.3 mm Na-GTP, pH adjusted to 7.2 with KOH (278–285 mOsmol). For spontaneous IPSCs, the pipette was filled with an internal solution containing 135 mm CsCl, 4 mm NaCl, 0.5 mm CaCl_2_, 10 mm HEPES, 5 mm EGTA, 2 mm Mg-ATP, 0.5 mm Na_2_-GTP, 10 mm QX-314, of which the pH had been adjusted to 7.2 with CsOH (278–285 mOsmol). During voltage-clamp experiments, neurons were clamped at either −70 or 0 mV to measure EPSCs or IPSCs, respectively. Whole-cell voltage-clamp recordings were performed using a MultiClamp 700B amplifier (Molecular Devices), filtered at 2 kHz, and digitized at 20 kHz using a Digidata 1550 digitizer (Molecular Devices).

##### FISH.

Mouse brains were extracted after anesthesia (Zoletil, 30 mg/kg, i.p). These mouse brains were cut into areas containing the somatosensory cortex regions, and the sections were dropped quickly into liquid N_2_ for instant freezing. The frozen brain tissue was embedded in an optimal cutting temperature compound block and kept overnight at −80°C in a deep freezer. The following day, the optimal cutting temperature block was transferred to a −20°C freezer for 2 h and cryosectioned into 10-μm-thick sections. FISH was performed using an RNAscope Multiplex Fluorescent Reagent Kit (Advanced Cell Diagnostics) according to the manufacturer's protocol. We used the probes designed by the manufacturer, including the RNAscope Probe-Mm-Sst, Mm-Vip, Mm-Npy, Mm-Nos1, and Mm-Gad1 (Advanced Cell Diagnostics). Confocal imaging was taken using a TCS SP8 confocal microscope (Leica Microsystems) using a 20× objective lens. Quantification was performed with ImageJ software.

##### Immunohistochemistry.

Mice were perfused through the heart with PBS, pH 7.4, followed by 4% PFA. Brains were removed from the skull, postfixed with 4% PFA for 12 h at 4°C, and then placed in a 30% sucrose solution at 4°C for 3 d. Using a cryomicrotome (CM 1950, Leica Microsystems), 40-μm-thick frozen coronal sections were collected in 0.1 m PBS. Sections were incubated in −20°C methanol for 10 min, washed in PBS, and incubated in a blocking solution (10% donkey serum in universal blocking solution, 00–8120, Invitrogen) for 1 h at room temperature. Next, sections were incubated in primary antibodies in PBS overnight at 4°C and washed in PBS 3 × 5 min. Then the sections were incubated in secondary antibodies for 2 h at room temperature and washed in PBS 3 × 5 min. Nuclear counterstaining was performed with 100 ng/ml DAPI solution (1:10,000) in PBS for 10 min. Primary antibodies were used at the following concentration: rabbit anti-nNOS (1:800, Millipore) and mouse anti-GAD67 (1:300, Millipore). Secondary antibodies conjugated with Alexa-488 (1:350, Invitrogen) and Alexa-568 (1:350, Invitrogen) were used to visualize the signals. Fluorescent images for cell counting were taken using a TCS SP8 confocal microscope (Leica Microsystems) using 20× objective lens. Quantification was performed with ImageJ and Imaris software (Bitplane, RRID:SCR_007370).

##### Pharmacological agents.

U46619 (no. 16450) was purchased from Cayman Chemical. TTX (ab120055) was purchased from Abcam. NMDA (M3262), AMPA hydrobromide (A9111), muscimol (M1523), baclofen (B5399), NBQX disodium salt hydrate (N183), SOM (S1763), and BIBP 3226 (B174) were purchased from Sigma-Aldrich. CGP 55845 hydrochloride (no. 1248), bicuculline methiodide (no. 2503), d-AP5 (no. 0106), and *N*^ω^-propyl-l-arginine hydrochloride (l-NPA, no. 1200) were purchased from Tocris Bioscience.

##### Statistical analyses.

All reported values are presented as mean ± SEM. All statistical analyses were performed using the SPSS statistical software (IBM, RRID:SCR_002865). Shapiro–Wilk tests were performed on all datasets to determine whether the data were normally distributed. Depending on the normality outcome, two-tailed independent-samples *t* tests or two-tailed Mann–Whitney *U* tests were used for comparison of the two groups. For datasets that compared more than two groups, the Kruskal–Wallis test or one-way ANOVAs with Bonferroni *post hoc* comparisons were used. The value of *p* < 0.05 was accepted as statistically significant.

## Results

### Validation of the mouse chronic stress model

**Figure 1.**
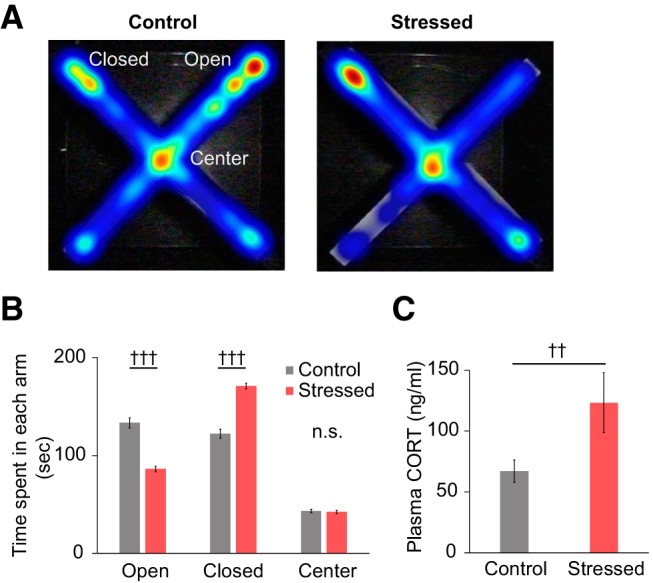
Behavioral and physiological validation of the chronic stress mouse model. ***A***, Representative heatmaps showing the exploration of mice during a 5 min EPM test. Each image was obtained from a single mouse. Red represents more time spent in an area. Blue represents less time spent in an area. ***B***, Average duration of time spent in each area of the EPM (control, *n* = 117; stressed, *n* = 123). ***C***, Comparison of baseline plasma corticosterone concentrations (control, *n* = 25; stressed, *n* = 25). Data are mean ± SEM. ^††^*p* < 0.01; ^†††^*p* < 0.001; n.s., not significant; Mann–Whitney *U* test.

Chronic restraint stress is a well-established protocol that induces anxiety-like behaviors in mice (Buynitsky and Mostofsky, 2009; Lee et al., 2015, 2018). The EPM, which contains both closed and open arms, is used to test the level of anxiety-like behaviors in animal subjects: the longer the time spent in the closed arms, the higher the level of anxiety-like behavior. In this study, we measured the duration of time both control and stressed mice spent in the open and closed arms of the EPM during a 5 min period. Chronically stressed mice exhibited a higher level of anxiety-like behavior compared with the control mice (stressed vs control; time spent in the closed arms: 171.40 ± 2.77 s vs 122.29 ± 4.74 s, *U*_(123, 117)_ = 3042.5, *p* < 0.001; open arms: 85.77 ± 2.67 s vs 133.41 ± 5.08 s, *U*_(123, 117)_ = 3176.5, *p* < 0.001, *U* test; Fig. 1*A*,*B*). We tested 186 stressed mice with EPM, and 63 mice (∼34% of stressed mice) showed stress resilience in our behavioral phenotyping results. This result is consistent with a previous study reporting that 30%–40% of chronically stressed mice show stress-resilient phenotype (Golden et al., 2011). We measured plasma corticosterone in blood samples taken from control and chronically stressed mice. Baseline corticosterone concentrations were elevated in the stressed group (stressed vs control: 123.31 ± 24.66 ng/ml vs 67.03 ± 9.18 ng/ml, *U*_(25, 25)_ = 166, *p* = 0.004, *U* test; Fig. 1*C*), indicating that HPA axis regulation was disrupted in those mice.

### Reduced hemodynamic response to forelimb stimulation in chronically stressed mice

**Figure 2.**
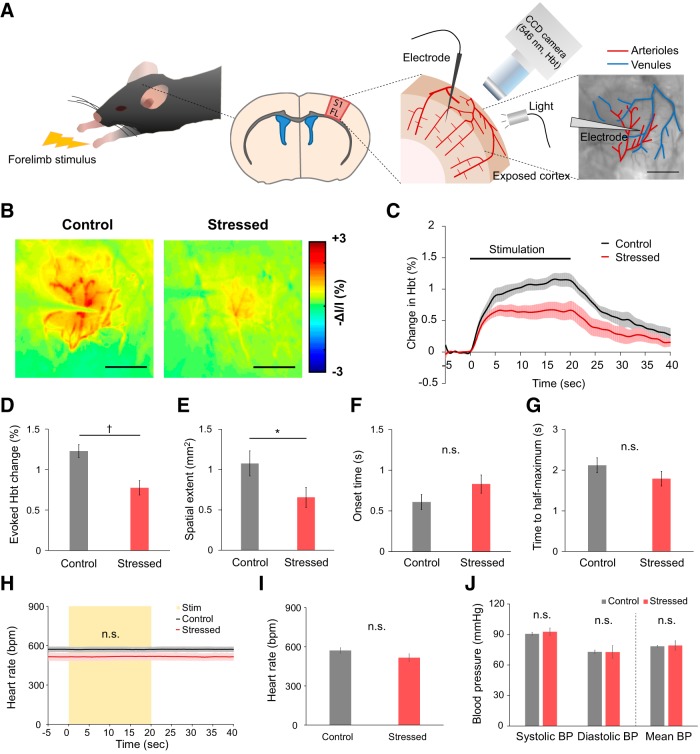
Reduced hemodynamic responses to forelimb stimulus in chronically stressed animals. ***A***, Experimental settings for *in vivo* OIS imaging. Light reflected from the exposed S1FL cortex (i.e., the forelimb region of the primary somatosensory cortex) was filtered at the 546 nm wavelength, which is a measure of the Hbt. To assess the NVC relationship during functional hyperemia, LFPs were measured simultaneously with the OIS imaging. ***B***, Representative Hbt-weighted OIS images during forelimb stimulation in control (left) and stressed mice (right). Each image represents the average of 18 trials from a single individual. Scale bars, 1 mm. ***C***, Time course traces of relative changes in the evoked Hbt signal following forelimb stimulation, relative to baseline levels (control, black, *n* = 10; stressed, red, *n* = 10). ***D***, Average of the maximum evoked Hbt change. ***E***, Comparison of the area of activated region (spatial extent) for the time point at which the maximum Hbt change was observed. ***F***, Time of onset. ***G***, Time to reach half-maximum of the first peak of the Hbt response. ***H***, Time course traces of heart rate monitored during OIS imaging sessions, including the sensory stimulation period (indicated with the yellow box). ***I***, Average of the heart rate measured throughout the experiments (control, *n* = 10; stressed, *n* = 10). ***J***, Blood pressure measured in urethane-anesthetized mice (control, *n* = 3; stressed, *n* = 3) using a tail-cuff blood pressure measurement system. Data are mean ± SEM. ^†^*p* < 0.05 (Mann–Whitney *U* test). *p < 0.05 (independent *t* test); n.s., not significant.

To assess hemodynamic response to sensory stimulation in the chronically stressed mice, we used OIS imaging. Due to the well-organized topographical representation of the body surface in the primary somatosensory cortex, sensory-evoked neurovascular response is widely studied with OIS and vascular imaging (Berwick et al., 2008; Devor et al., 2008; Hillman, 2014; Iordanova et al., 2015; Cai et al., 2018). Thus, to study hemodynamic response, forelimb stimulation was recorded by OIS imaging of the forelimb activation region (S1FL) of the primary somatosensory cortex (Fig. 2*A*).

Since the stimulation-induced increase in Hbt results in a reduction of the reflected 546 nm OIS intensity (increase in absorption), Hbt-weighted OIS images taken during forelimb stimulation are presented as inverted values of relative change (Fig. 2*B*). Higher changes (red) indicate larger increases in local CBV under the assumption that hemoglobin concentration remains constant. Overall, chronically stressed mice showed significantly reduced stimulus-evoked changes in Hbt. First, peak Hbt response was significantly lower in the chronically stressed group (stressed vs control: 0.78 ± 0.09% vs 1.23 ± 0.08%, *U*_(10, 10)_ = 16, *p* = 0.010, *U* test; Fig. 2*C*,*D*). Second, the spatial extent of the stimulus-evoked Hbt response was smaller in the stressed animals (stressed vs control: 0.66 ± 0.12 mm^2^ vs 1.08 ± 0.16 mm^2^, *t*_(18)_ = 2.109, *p* = 0.049, *t* test; Fig. 2*E*). Additionally, the onset of the Hbt response tended to be delayed in the stressed group (stressed vs control: 0.83 ± 0.11 s vs 0.61 ± 0.09 s, *t*_(18)_ = 0.841, *p* = 0.146, *t* test; Fig. 2*F*), and the time to half-maximum of the peak was slightly less in the stressed animals (stressed vs control: 1.79 ± 0.18 s vs 2.12 ± 0.18 s, *t*_(18)_ = 1.282, *p* = 0.216, *t* test; Fig. 2*G*); however, neither difference was statistically significant.

Cardiovascular physiological parameters were measured throughout these experiments since the altered hemodynamic responses in the stressed group may be due to changes in cardiovascular physiology. Both control and chronically stressed mice maintained a normal range of physiological parameters under urethane anesthesia. There was no stimulation-induced change in heart rate and no significant difference between the two groups (stressed vs control: 515.71 ± 30.27 bpm vs 571.32 ± 20.36 bpm, *t*_(18)_ = 1.525, *p* = 0.145, *t* test; Fig. 2*H*,*I*). In addition, there was no difference in mean blood pressure (stressed vs control: 79.2 ± 2.71 mmHg vs 78.47 ± 0.49 mmHg, *U*_(3, 3)_ = 3, *p* = 0.513, *U* test; Fig. 2*J*). It is evident that chronic stress does not alter cardiovascular effects under our experimental conditions.

Summarily, chronic restraint stress decreased hemodynamic response to forelimb stimulation. Moreover, these results obtained with a mouse model are consistent with our previous work using a rat model of chronic restraint stress, which also showed reduced hemodynamic response to sensory stimuli (Lee et al., 2015).

### Neural response to forelimb stimulation was attenuated in chronically stressed conditions

**Figure 3.**
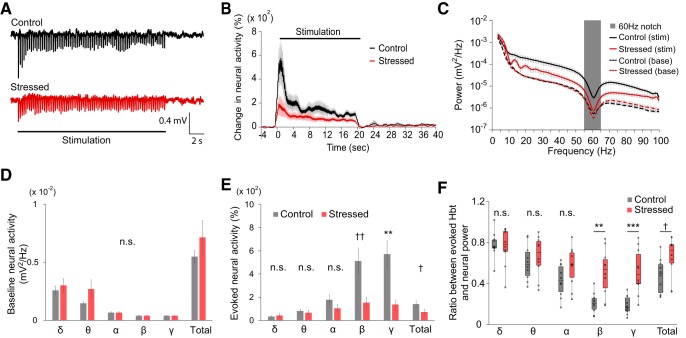
Reduced neural response to forelimb stimulus and alteration of the NVC relationship in chronically stressed animals. ***A***, Representative raw LFP traces acquired from control and stressed mice. ***B***, Time course traces of relative changes in the evoked neural activity following forelimb stimulation, relative to prestimulus baseline levels (control, black, *n* = 10; stressed, red, *n* = 10). ***C***, Power spectra of LFP recorded during stimulation (solid lines; control group in black and stressed group in red) and prestimulus baseline (dashed lines) periods. Gray box represents the frequency range (55–65 Hz) excluded from all analyses due to use of a 60 Hz notch filter. ***D***, Comparison of baseline neural activity in different frequency ranges. ***E***, Comparison of the evoked neural activity in different frequency ranges. ***B–E***, Data are mean ± SEM. ***F***, Box plot (box-and-whisker diagram) of the ratio of the evoked Hbt response to the evoked neural response. ^†^*p* < 0.05; ^††^*p* < 0.01; Mann–Whitney *U* test. **p < 0.01; ***p < 0.001; independent-samples *t* test; n.s., not significant.

Simultaneously with OIS imaging, we also measured LFP in the somatosensory cortex to investigate whether reduced hemodynamic response to stimuli is associated with any changes in evoked neural activity (Fig. 2). LFP in the forelimb region was measured at a depth of ∼300 μm, targeting layer II/III of the somatosensory cortex. Before insertion of the LFP recording electrode, a preliminary forelimb stimulation was performed, and the recording site was selected to be the center of the stimulus-evoked hemodynamic response, avoiding large surface vessels (Fig. 2*A*). LFP traces in Figure 3*A* represent the average of 18 trials recorded during stimulation of 1 control and 1 stressed animal, respectively.

A comparison of evoked neural response amplitude revealed that stressed mice showed a markedly reduced relative increase of LFP power during the 20 s stimulation period (Fig. 3*B*). In other words, evoked neural responses were significantly suppressed in the chronically stressed animals (stressed vs control: 72.70 ± 23.85% vs 140.22 ± 28.81%, *U*_(10,10)_ = 19, *p* = 0.019, *U* test; data named as “Total” in Fig. 3*E*), whereas spontaneous neural activities were not significantly different (Fig. 3*C*,*D*). A power spectrum analysis of the stimulus-evoked LFP signal revealed significant suppression in the higher-frequency bands (stressed vs control; β band: 152.81 ± 48.20% vs 512.55 ± 111.77%, *U*_(10, 10)_ = 12, *p* = 0.004, *U* test; γ: 137.65 ± 39.96% vs 572.98 ± 119.22%, *t*_(10.997)_ = 3.462, *p* = 0.005, *t* test), whereas the power in the lower-frequency bands remained unchanged (Fig. 3*C*,*E*). In light of these results, it is evident that reduced hemodynamic response to forelimb stimulus is likely to be associated with the attenuation of evoked neural activity in higher-frequency bands.

### Chronic stress-induced alteration in the NVC relationship

Although both neural and hemodynamic responses to forelimb stimuli are reduced in chronically stressed conditions, there is a possibility that the NVC relationship itself is unaffected. Therefore, we assessed whether the NVC relationship had been altered by calculating the ratio of the evoked hemodynamic response to the evoked neural response. The calculation of the ratio between evoked neural activity and subsequent hemodynamic response was based on studies that assessed NVC relationships in the cortical regions. The broad consensus from those studies is that there is a positive linear relationship between ongoing neural activity and the subsequent hemodynamic responses in the normal brain (Niessing et al., 2005; Devonshire et al., 2012; Iordanova et al., 2015; Lecrux et al., 2017). In chronically stressed mice, the ratio between the peak value of Hbt changes (Fig. 2*D*) and the evoked neural activity (Fig. 3*E*) was significantly changed in the beta and gamma frequency bands as well as over the total range of frequencies (2–100 Hz) (stressed vs control; β band: 0.51 ± 0.07 vs 0.21 ± 0.03, *t*_(12.335)_ = −3.862, *p* = 0.002, *t* test; γ band: 0.52 ± 0.07 vs 0.19 ± 0.03, *t*_(11.857)_ = −4.413, *p* = 0.0009, *t* test; total neural activity: 0.66 ± 0.06 vs 0.48 ± 0.05, *U*_(10, 10)_ = 19, *p* = 0.019, *U* test; Figure 3*F*). In summation, chronic stress altered the NVC relationship during functional hyperemia.

### Vascular dynamics triggered by neuronal activation were altered in acute brain slices of chronically stressed mice

From *in vivo* data, we hypothesized that altered NVC may have been caused by the dysfunction of specific cellular signaling that controls vascular responses. To elucidate which cell signals were primarily affected, we used acute brain slices, which allow better accessibility to measure complex signaling between neurons and blood vessels. During all vascular imaging experiments using the acute brain slices, we added the arteriole preconstrictor, U46619 (65–100 nm, Cayman Chemical) to the bath to induce vascular tone (Cauli et al., 2004; Gordon et al., 2008; Longden et al., 2014; Institoris et al., 2015). Within each slice, we selected 8- to 14-μm-diameter penetrating arterioles with thick smooth muscle in layer II/III of the somatosensory cortex.

**Figure 4.**
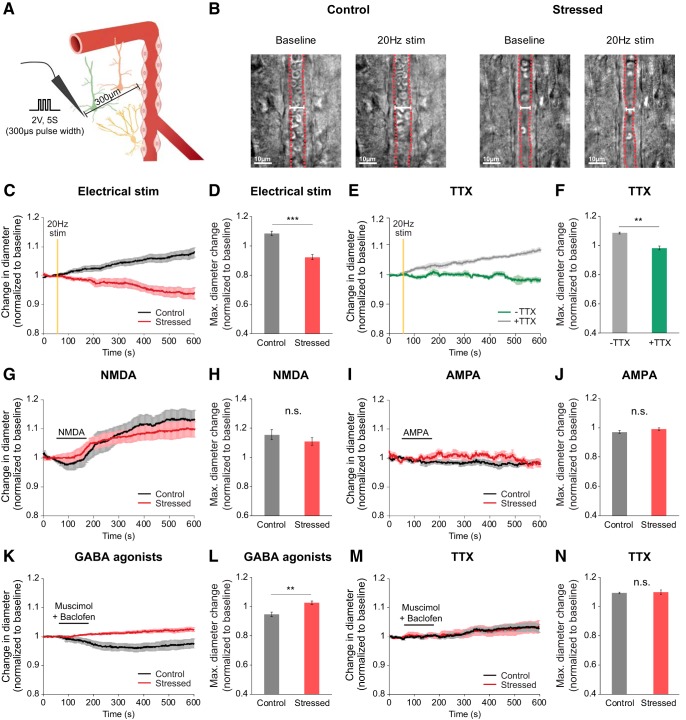
Altered vascular dynamics in chronically stressed mice, as measured in acute brain slices. ***A***, The schematic setup showing the experiment. ***B***, Representative images of baseline and postelectrical 20 Hz stimulation arterioles in the somatosensory cortex of control and stressed mice. Red vertical lines were traced along vascular walls for visualization of penetrating arterioles. ***C***, ***D***, Time course traces and maximal relative amplitudes of arteriolar diameter changes following electrical stimulation for control (black, *n* = 10) and stressed brain (red, *n* = 11). Clearly, stimulation induces dilation for the control, but constriction for the stressed brain slice. ***E***, ***F***, Time course traces and maximal relative amplitudes of vascular diameter changes after stimulation in the presence (green, *n* = 6) or absence of TTX (gray, *n* = 5) in the control mouse brain slice. In the control brain slice, stimulation induces vascular dilation (1.08 ± 0.01), which is abolished by TTX application (0.98 ± 0.01, *U*_(6,5)_ = 0_,_ *p* = 0.004, *U* test). ***G–J***, Time course traces and maximal relative amplitudes of vessel diameter change induced by 2 min application (horizontal black line) of 30 μm NMDA (***G***, ***H***; control, *n* = 9; stressed, *n* = 7) and 10 μm AMPA (***I***, ***J***; control, *n* = 5; stressed, *n* = 5) for control (black) and stressed brain slice (red). Both NMDA and AMPA mimic the action of glutamate. Neither compounds produce any significant difference in vascular diameter for control versus stressed slice. ***K–M***, Time course traces (***K***, ***M***) and maximal relative amplitudes (***L***, ***N***) of vessel diameter change induced by 2 min treatment of GABA receptor agonists (100 μm muscimol + 50 μm baclofen) in the absence (***K***, ***M***) and presence of TTX (***L***, ***N***) for control (black, *n* = 13 without and 8 with TTX) and stressed brain slices (red, *n* = 11 without and 11 with TTX). Data are mean ± SEM. **p < 0.01, ***p < 0.001, n.s., not significant.

First, we tested whether chronic stress alters vessel response to electrical stimulation. A concentric bipolar electrode was positioned 300 μm away from the arteriole of interest to evoke neuronal excitation (Institoris et al., 2015). Then, we recorded the responses of the arteriole following 20 Hz electrical stimulation (300 μs, 2 V, 5 s) (Fig. 4*C*). In each group, both vasodilation and vasoconstriction were observed; however, vasoconstriction was more prevalent in chronically stressed mice (maximal diameter change vs baseline: 0.92 ± 0.02), whereas vasodilation was observed more often in the control mice (1.08 ± 0.02, *U*_(11,10)_ = 2, *p* = 0.00019, *U* test; Fig. 4*D*). Second, the same electrical stimulation experiments were repeated, but with the addition of 1 μm tetrodotoxin (TTX). Use of this neurotoxin allowed us to determine whether these vascular responses were evoked by direct electrical stimulation to vessel or by neural activation. In the presence of TTX, there was almost no vascular response following 20 Hz electrical stimulation in control mice (TTX: 0.98 ± 0.01; Fig. 4*E*,*F*). These results indicate that the differences in vascular responses between normal and stressed mice are a consequence of differences in neuronal activity.

After confirming that the altered vascular responses observed in chronically stressed mice was mediated by neuronal activity, we used various receptor agonists and antagonists to explore whether these neuronal changes originate from either the glutamatergic or GABAergic pathways. We then assessed the implication of NMDA receptors, which elicit the vasodilatory pathway via activation of the COX-2 enzyme (Gordon et al., 2008; Lecrux and Hamel, 2011; Lacroix et al., 2015); 30 μm NMDA application elicited arteriole vasodilation to the same degree in both stressed and control mice (stressed vs control mice: 1.11 ± 0.03 vs 1.16 ± 0.03, *U*_(7,9)_ = 21_,_ *p* = 0.266, *U* test; Fig. 4*G*,*H*). We also tested the implication of AMPA receptors, which has also been suggested to be a mediator of cerebral vasodilation via activation of a subsequent adenosine pathway (Ohata et al., 2006); 10 μm AMPA application did not evoke vasodilation in either group (stressed vs control mice: 0.99 ± 0.01 vs 0.97 ± 0.01, *U*_(5,5)_ = 7_,_ *p* = 0.251, *U* test; Fig. 4*I*,*J*). After confirming that NMDA-mediated vasodilation remains undamaged under chronically stressed conditions, we tested a GABAergic pathway of vascular response via GABA receptor agonists. GABA receptor agonist (100 μm muscimol + 50 μm baclofen) stimulation induced dramatically dissimilar responses in control and stressed mice. While considerable arteriolar vasoconstriction occurred in control mice (0.95 ± 0.02), some vasodilation was observed instead in chronically stressed mice (1.03 ± 0.01, *U*_(13,11)_ = 20, *p* = 0.003, *U* test; Fig. 4*K*,*L*). There is also a possibility of a direct effect of GABA receptors on blood vessels. However, TTX abolished GABA agonist-mediated constriction, GABA receptor agonists did not act directly on blood vessels (stressed vs control mice: 1.05 ± 0.02 vs 1.04 ± 0.01, *t*_(12.06)_ = −0.33, *p* = 0.75, *t* test; Fig. 4*M*,*N*). Consequently, NMDA-mediated vascular response is intact under chronic stress, whereas GABA-mediated vascular response is altered under chronic stress.

### Diminished frequency of IPSCs from pyramidal cells in chronically stressed mice

**Figure 5.**
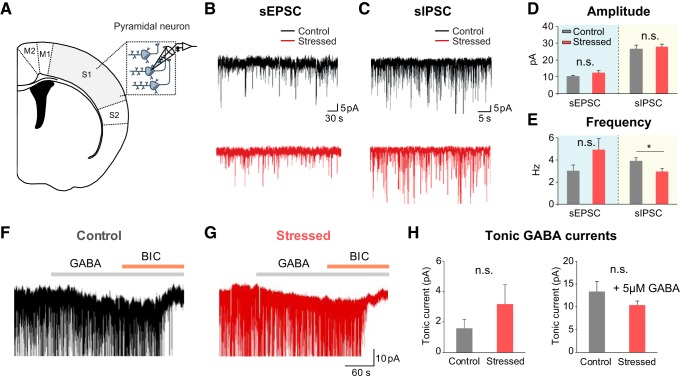
Reduction of the frequency of sIPSCs from pyramidal cells in chronically stressed mice. ***A***, The schematics showing patch-clamp recording from pyramidal cells in somatosensory cortex. ***B***, ***C***, Representative traces of EPSCs (***B***) and IPSCs (***C***) in control (top, black) and stressed mice (bottom, red). ***D***, The amplitude of sEPSCs (control: *n* = 10; stressed: *n* = 11) and sIPSCs (control: *n* = 10; stressed: *n* = 13). ***E***, The frequency of sEPSCs (control: *n* = 10; stressed: *n* = 11) and sIPSCs (control: *n* = 10; stressed: *n* = 13). ***F***, ***G***, Representative traces of tonic GABA_A_R currents in control (left, black) and stressed mice (right, red). ***H***, The amplitude of tonic GABA_A_R currents measured in two groups (control: *n* = 10; stressed: *n* = 11) and the amplitude of tonic GABA_A_R currents measured with treatment of 5 μm GABA in two groups (control: 13.28 ± 2.26 pA, *n* = 9; stressed: 12.3 ± 2.13, *n* = 10; *U*_(9,10)_ = 38, *p* = 0.567, *U* test). Data are mean ± SEM. *p < 0.05, n.s., not significant.

Examining the electrophysiological properties of pyramidal cells through whole-cell patch-clamp recording allowed us to confirm whether synaptic transmission was altered under chronic stress conditions. Measurements of spontaneous excitatory postsynaptic currents (sEPSCs) from neurons in the somatosensory cortex showed that sEPSC amplitude (stressed vs control mice: 12.12 ± 1.24 vs 9.85 ± 0.74 pA, *U*_(11,10)_ = 34, *p* = 0.139, *U* test) and frequency (4.90 ± 0.97 vs 3.03 ± 0.53 Hz, *t*_(19)_ = −1.649, *p* = 0.116, *t* test) had an increasing trend, albeit not significantly different, between the two groups (Fig. 5*B* and Fig. 5*D*,*E*). However, the frequency of spontaneous inhibitory postsynaptic currents (sIPSCs) (stressed vs control mice: 2.88 ± 0.30 vs 3.85 ± 0.32 Hz, *t*_(21)_ = 2.204_,_ *p* = 0.039, *t* test) was significantly reduced in chronically stressed mice with the sIPSC amplitude remaining unchanged (27.74 ± 1.62 vs 26.43 ± 2.06 pA, *t*_(21)_ = −0.508_,_ *p* = 0.617, *t* test; Fig. 5*C* and Fig. 5*D*,*E*). Furthermore, we measured tonic GABA currents that are mainly mediated by the extrasynaptic GABA_A_ receptor, whose activity has bidirectional effects on hyperemia (Jessen et al., 2015). The amplitude of tonic GABA currents (stressed vs control mice: 4.94 ± 2.14 vs 1.58 ± 0.59 pA, *U*_(12,10)_ = 47.5_,_ *p* = 0.405, *U* test) was not notably different between the two groups (Fig. 5*F–H*). Collectively, these observations suggest that chronic stress disrupts presynaptic GABA release evoked by neural activity and changes the balance of excitatory and inhibitory currents (E/I balance).

### Dysfunction of GABA_A_ receptors contributes to NVC impairment in chronically stressed mice

**Figure 6.**
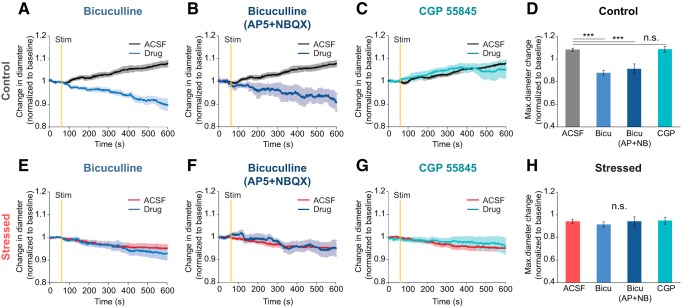
Alteration of GABA_A_ receptor-mediated vascular responses induced by 20 Hz focal electrical stimulation in stressed mice. ***A–C***, ***E–G***, Time course traces of arteriolar diameter change in control (***A–C***) and stressed brain tissues (***E–G***) exposed to two conditions: under ACSF circulation (black or red) or with treatment of drug (blue); 100 μm bicuculline, a GABA_A_ antagonist (***A***, ***E***), 100 μm bicuculline with glutamergic antagonists (10 μm AP5 + 5 μm NBQX) (***B***, ***F***), and 10 μm CGP 55845, a GABAB antagonist (***C***, ***G***) were used as drug circulation. ***D***, Maximum diameter change amplitude in the control group following each drug treatment (ACSF: *n* = 10; bicuculline: *n* = 8; bicuculline with AP5 and NBQX: *n* = 5; CGP 55845: *n* = 7). ***H***, Maximum diameter change amplitude in the chronically stressed group following each drug treatment (ACSF: *n* = 11; bicuculline: *n* = 8; bicuculline with AP5 and NBQX: *n* = 5; CGP 55845: *n* = 7). Data are mean ± SEM. ***p < 0.001, n.s., not significant. Statistical significance was tested with the one-way ANOVA followed by Bonferroni *post hoc* tests according to the results of a normal distribution test (Shapiro–Wilk test).

GABA, which is a major inhibitory neurotransmitter, is modulated via GABA_A_ and GABA_B_ receptors. GABA_A_ is a ligand-gated ion receptor with a chloride ion-selective pore, whereas GABA_B_ is a G-protein-coupled receptor. Each GABA receptor type has been reported to affect NVC (Fergus and Lee, 1997; Jessen et al., 2015). We differentiated between the two GABA receptors' impact on NVC in chronically stressed mice by using either a GABA_A_ receptor antagonist (bicuculline, 100 μm) or a GABA_B_ receptor antagonist (CGP 55845, 20 μm). Each antagonist was applied for the 20 min preceding 20 Hz focal electrical stimulation and maintained throughout the experiment. In control mice, selective GABA_A_ receptor blockade via bicuculline-induced vasoconstriction following electrical stimulation (ACSF: 1.08 ± 0.01, bicuculline: 0.88 ± 0.22, *p* < 0.001; Fig. 6*A*,*E*), whereas selective GABA_B_ receptor blockade with CGP 55845 produced no significantly different responses in vascular dynamics (CGP 55845: 1.09 ± 0.01, *p* = 1; Fig. 6*C*,*G*). To determine whether the vascular responses caused by bicuculline in the control group was independent from glutamatergic signaling, we performed the same bicuculline experiment while blocking NMDA and AMPA receptors. Since the experimental results under receptor blockade did not prove to differ, it is apparent that vascular responses triggered by bicuculline are likely to be independent to glutamatergic signaling (bicuculline with AP5 and NBQX: 0.92 ± 0.04, *F*_(3,26)_ = 26.708, *p* < 0.001, one-way ANOVA with Bonferroni *post hoc* test; Fig. 6*B*,*F*). In stressed mice, similar vascular responses were shown regardless of drug application contrasting with the results obtained from the controls (ACSF: 0.94 ± 0.02, bicuculline: 0.91 ± 0.02, *p* = 1, bicuculline with AP5 and NBQX: 0.94 ± 0.04, *p* = 1, CGP 55845: 0.95 ± 0.03, *F*_(3,27)_ = 0.386, *p* = 1, one-way ANOVA with Bonferroni *post hoc* test; Fig. 6*B*,*D*). Ultimately, our observations corroborate the prominence of the role of GABA_A_ receptor metabolites in maintaining normal NVC.

### Impaired neuronal NOS-mediated NVC in chronically stressed mice

**Figure 7.**
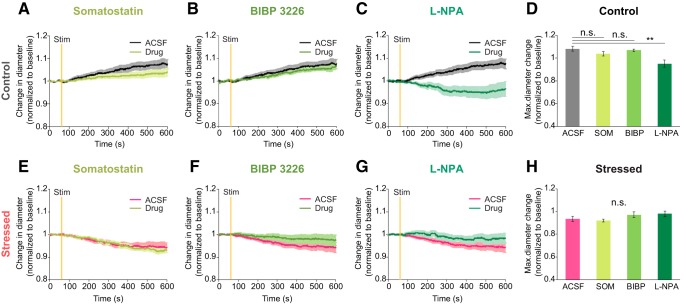
Alteration of nNOS-mediated vascular responses induced by 20 Hz focal electrical stimulation in stressed mice. ***A–C***, ***E–G***, Time course traces of arterial diameter change in control (***A–C***) and stressed brain tissues (***E–G***) under ACSF (black or red) or with drug treatment (green). Drug was 1 μm SOM (***A***, ***E***), a neuropeptide that agonist for SOM, 1 μm BIBP 3226 (***B***, ***F***), an antagonist for the neuropeptide Y receptor, and 10 μm l-NPA (***C***, ***G***), a nNOS inhibitor. ***D***, Maximum diameter change amplitude following each drug treatment in the control group (ACSF: *n* = 10; SOM: *n* = 13, BIBP 3226: *n* = 6, l-NPA: *n* = 10). ***H***, Maximum diameter change amplitude following each drug treatment in the chronically stressed group (ACSF: *n* = 11; SOM: *n* = 12, BIBP 3226: *n* = 5; l-NPA: *n* = 12). Data are mean ± SEM. **p < 0.01, n.s., not significant. A one-way ANOVA followed by Bonferroni *post hoc* tests was used for analysis of a drug treatment effect in the control group, and the Kruskal–Wallis test was used for statistical analysis of the chronically stressed group according to the results of a normal distribution test (Shapiro–Wilk test).

Different subtypes of GABAergic interneurons control vasodilation by releasing nitric oxide (NO) and vasoactive intestinal peptide (VIP). Some subtypes also control vasoconstriction by releasing the neuropeptides, such as SOM and NPY (Cauli et al., 2004; Uhlirova et al., 2016). To evaluate which GABAergic interneurons create dysfunctional hemodynamics in chronically stressed animals, we investigated NVC under specific GABAergic interneurons' subtype agonists and antagonists. Specific GABAergic interneurons' subtype agonists and antagonists were applied for the 20 min preceding 20 Hz focal electrical stimulation and maintained throughout the experiment. When the drug, SOM, was applied, the vascular response to local electrical stimulus was not altered compared with ACSF conditions in either the control (ACSF: 1.08 ± 0.02, SOM: 1.04 ± 0.02, *p* = 0.981; Fig. 7*A*) or the stressed group (ACSF: 0.93 ± 0.02, SOM: 0.92 ± 0.01; Fig. 7*E*). Thus, SOM-expressing interneurons have less relevance to alteration of NVC evoked by chronic stressors. Like SOM, selective NPY Y1 receptor blockade with 1 μm BIBP 3226 also had little effect on vascular response compared with the ACSF condition in either the control group (BIBP 3226: 1.07 ± 0.01, *p* = 1; Fig. 7*B*) or the stressed group (BIBP 3226: 0.97 ± 0.02; Fig. 7*F*). In contrast, vasoconstriction was strongly induced in the control group <10 μm l-NPA, which is a selective nNOS receptor blocker (l-NPA: 0.95 ± 0.03, *F*_(3,35)_ = 5.952, *p* = 0.002, one-way ANOVA with Bonferroni *post hoc* test; Fig. 7*C*). nNOS is one of the isomers of the enzyme involved in the synthesis of NO, which is a major vasodilator that regulates CBF (Cauli and Hamel, 2010; Kilduff et al., 2011). Unlike the control group, the stressed mice showed similar vascular response, even in the presence of l-NPA (m-NPA: 0.98 ± 0.02, χ^2^_(3)_ = 5.432, *p* = 0.143, Kruskal–Wallis test; Fig. 7*G*) Therefore, the nNOS-mediated vasodilation pathway is likely to be impaired by chronic stress.

### Reduced numbers of nNOS-expressing GABAergic interneurons in chronically stressed mice

**Figure 8.**
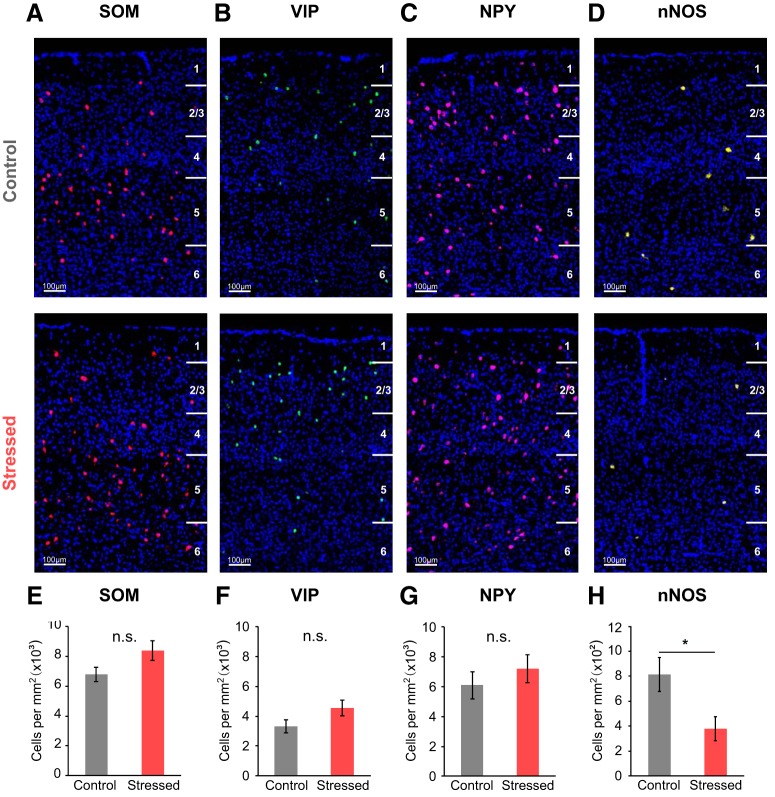
Decreased mRNA expression of nNOS in the somatosensory cortex of chronically stressed mice. ***A***, ***E***, SOM mRNA expression in the somatosensory cortex of both control and chronically stressed mice (control: *n* = 7; stressed: *n* = 5). ***B***, ***F***, VIP mRNA expression (control: *n* = 7; stressed: *n* = 5). ***C***, ***G***, NPY mRNA expression (control: *n* = 7; stressed: *n* = 5). ***D***, ***H***, nNOS mRNA expression (control: *n* = 5; stressed: *n* = 5). Data are mean ± SEM. *p < 0.05 (Mann–Whitney *U* test); n.s., not significant.

After confirming the importance of the role of nNOS-expressing neurons in NVC, we checked the mRNA expression of several types of GABAergic interneurons in both control and chronically stressed mice by using FISH. Only the mRNA expression of nNOS was significantly reduced in chronically stressed mice (stressed vs control mice: 379.56 ± 95.65 vs 815.37 ± 137.59 cells/mm^2^, *U*_(5,5)_ = 3, *p* = 0.047, *U* test; Fig. 8*D*,*H*). By comparison, the mRNA expression of SOM (stressed vs control mice: 8388.89 ± 645.69 vs 6791.69 ± 465.60 cells/mm^2^_,_ *U*_(5,7)_ = 8, *p* = 0.123, *U* test), VIP (4540.36 ± 522.48 vs 3316.69 ± 429.27 cells/mm^2^, *U*_(5,7)_ = 6, *p* = 0.062, *U* test), and NPY (7207.65 ± 946.98 vs 6110.79 ± 907.24 cells/mm^2^, *U*_(5,7)_ = 12, *p* = 0.372, *U* test) appeared to have no differences between the two groups (Fig. 8).

**Figure 9.**
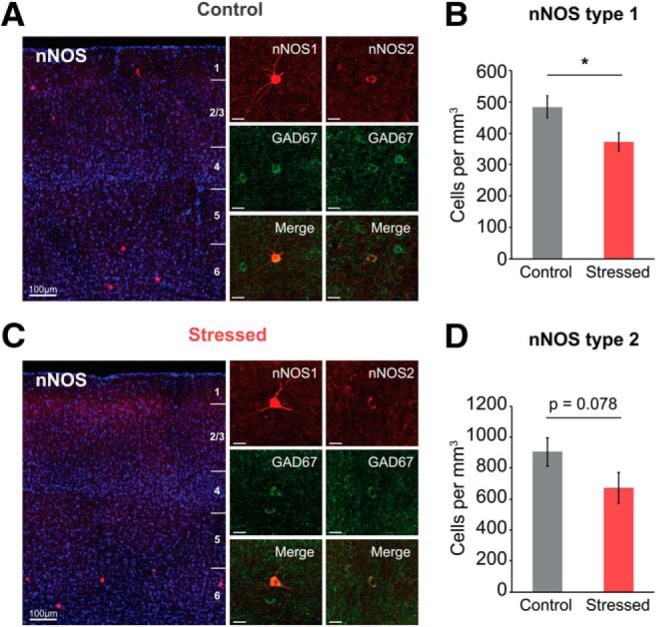
Decreased protein expression of nNOS-expressing neurons in the somatosensory cortex of chronically stressed mice. ***A***, ***C***, Cortical layer-dependent (left) and expanded IHC images (right) of nNOS-expressing neurons from control and chronically stressed mice. Type 1 nNOS-expressing neurons have large soma with strong IHC intensity, whereas Type 2 nNOS neurons have small soma with weak intensity. Scale bars: subplots, 20 μm. ***B***, ***D***, The number of Type 1 (control: *n* = 12; stressed: *n* = 11) and Type 2 nNOS neurons (control: *n* = 6; stressed: *n* = 6). Data are mean ± SEM. *p < 0.05, Mann–Whitney *U* test.

There are two classes of nNOS-expressing neurons: Type 1 and Type 2. Type 1 nNOS neurons show large somata with strong IHC intensity, whereas Type 2 nNOS neurons have small somata and weak intensity (Yan and Garey, 1997; Perrenoud et al., 2012). Type 1 nNOS neurons are predominantly located in deeper layers of the cortex and coexpress SOM, NPY, and PV. Conversely, Type 2 nNOS neurons are mainly located in layer II/III and VI and coexpress PV, SOM, and VIP (Magno et al., 2012; Perrenoud et al., 2012). Hence, we further confirmed the expression of each type of nNOS protein level using IHC. As a result, Type 1 nNOS neurons were considerably reduced in chronically stressed mice (stressed vs control mice: 372.08 ± 29.39 vs 492.11 ± 33.50 cells/mm^3^, *U*_(11,12)_ = 27, *p* = 0.016, *U* test; Fig. 9*B*). Differences in Type 2 nNOS neurons, while statistically insignificant, were apparently lower in chronically stressed mice (671.95 ± 101.13 vs 903.50 ± 91.83 cells/mm^3^, *U*_(6,6)_ = 7, *p* = 0.078, *U* test; Fig. 9*D*). Thus, it is difficult to entirely rule out the possibility that Type 2 nNOS neurons are unaffected by chronic stress. These results suggest that Type 1 nNOS neurons have more vulnerability to chronic stressor than Type 2 nNOS neurons. Collectively, our experimental outcomes imply that the reduction of nNOS expression contributes to the nNOS-mediated NVC impairment found in chronically stressed mice. nNOS-expressing neurons also coexpress SOM and other neuropeptides, such as NPY, which are known to be vasoconstrictors. Indeed, SOM seems to induce slight vasoconstriction trends in control mice, and NPY receptor blockade also seems to induce slight vasodilation trends in stressed mice (Fig. 7). Thus, future experiments using cell type-specific modulation by optogenetic or chemogenetic technique would be required to address that the exact role of GABAergic interneurons in controlling CBF in case they coexpress neuropeptides known as different vasomodulatory action.

## Discussion

This study demonstrates that NVC is disrupted during functional hyperemia in chronically stressed animals. The purpose of this study was to investigate both *in vivo* and *ex vivo* the underlying cellular mechanisms that drive such alterations. We compared sensory stimulus-evoked hemodynamic and neural responses in unstressed and stressed mice. These comparisons revealed an altered relationship between gamma frequency activity and hemodynamic response during forelimb stimulation in chronically stressed animals. Utilizing *ex vivo* vascular imaging along with pharmacological tests, patch-clamp recording, and FISH techniques, several key results emerged. First, there was a decrease in sIPSC frequency in chronically stressed mice. Second, GABA_A_ receptor and nNOS-mediated vascular responses are altered by chronic stress. Third, a reduced number of nNOS-expressing cells was observed in chronically stressed animals. Overall, our study clearly reveals that chronic stress has highly negative impacts on NVC, disrupting proper function of nNOS-expressing neurons.

### Neuronal origin of altered NVC under chronically stressed condition

In our *in vivo* data, chronic stress was associated with both attenuated hemodynamic response and LFP response to forelimb stimulus. Logothetis (2008) described the alteration in hemodynamic signal when the E/I balance changes. Changes in neural activity while maintaining the E/I balance would lead to proportional changes in hemodynamic response. Adopting this idea, if the chronic stress-associated reduction in hemodynamic response is directly proportional to the attenuated neural response, the reduced overall power of neural activity would be likely responsible for the decreased hemodynamic response. Results in Figure 3*F*, however, show that the NVC relationship was changed in chronically stressed mice. Thus, our interpretation of these data is that an imbalance between the excitatory and inhibitory cortical microcircuits is associated with chronic stress, and there is likely to be an additional component other than the reduction in net excitation during sensory stimulation to account for the reduction in hemodynamic response.

The results of our patch-clamp and LFP recordings suggest that the additional component that contributes to the altered NVC relationship may be dysfunctional inhibitory neuronal activity. Reduction in sIPSC frequency in the stressed brain (Fig. 5*E*) implies that GABA release from presynapses is decreased under chronic stress. Previous reports showed similar outcomes in terms of GABAergic interneuron activity. Under the chronically stressed condition, GABAergic inhibition was attenuated and the number of GABAergic interneurons was reduced (Czéh et al., 2015, 2018; Csabai et al., 2018). Since inhibitory neural circuits in the neocortex precisely control the E/I balance (Tremblay et al., 2016), change in sIPSC can modulate the E/I balance. Since a balanced interaction between excitation and inhibition generates gamma oscillation (Buhl et al., 1998), a modulation in the E/I balance can be reflected as altered gamma activity (Levin and Nelson, 2015). Here, we observed a selective reduction in the evoked gamma activity under chronic stress (Fig. 3*E*). Reports that emphasize the role of GABAergic interneurons on generating gamma oscillations (Cardin et al., 2009) also support that chronic stress exposure may differently affect excitatory and inhibitory cortical networks. Since evoked gamma activity during functional hyperemia correlates better with hemodynamic signal in the neocortex compared with lower-frequency activity (Niessing et al., 2005), selective attenuation in gamma activity may contribute to the decrease in hemodynamic response. Together, chronic stress induces an E/I imbalance in cortical microcircuits by affecting inhibitory neuronal activity, which may lead to reduction in gamma activity and hemodynamic response. These results suggest that the alteration of NVC under chronic stress originates from neuronal alteration.

### Contribution of glutamatergic neurons for altered NVC in chronically stressed mice

The activation of NMDA receptors in cortical excitatory neurons releases COX-2 products, which is followed by hemodynamic response (Gordon et al., 2008; Lecrux and Hamel, 2011; Lacroix et al., 2015). Hence, we sought to determine the implications of excitatory neuron activity on vascular response using acute brain slices. In this study, NMDA application induced vasodilation, consistent with a prior study (Lacroix et al., 2015), but a similar degree of vasodilation was shown in both control and stressed mice. This suggests that NMDA-mediated vascular responses remain intact under chronically stressed conditions. mGluRs are expressed in both reactive glia and neuronal cells, and controversy remains regarding their role in functional hyperemia. It has been suggested that the blockade of Group I mGluRs reduces evoked CBF responses to whisker stimulation (Lecrux et al., 2011). However, another group has shown that the blockade of mGluR5 had no impact on evoked hemodynamic changes (Calcinaghi et al., 2011). Consequently, definitive evidence on the mGluRs' role in functional hyperemia is still lacking and remains to be determined.

### Role of each GABA receptor on altered NVC in chronically stressed mice

Evoked CBF response to sensory stimulation recruits cortical excitatory neurons and inhibitory neurons; and, interestingly, GABA interneurons account for a large portion of c-Fos-positive neurons following sensory stimulation (Lecrux et al., 2011). Prior studies showing that optogenetic stimulation of vesicular GABA transporter evoked CBF increases (Anenberg et al., 2015; Vazquez et al., 2018) also support the crucial role of GABAergic interneurons on NVC. In the present study, we also observed that GABA receptor agonists induced significant differences between the control and stressed groups, indicating a strong correlation between chronic stressors and GABA-mediated vascular response. The vasoconstriction response following GABA agonist treatment was observed in the control group, which is consistent with prior demonstrations that high concentrations of the GABA_A_ receptor agonist muscimol attenuate CBF changes (Mathiesen et al., 2011) and GABA_B_ receptor agonists induce vasoconstriction in the hippocampus *in vitro* (Fergus and Lee, 1997). To more fully explore the specific role of each GABA receptor on vascular response, we applied blockers of GABA_A_ and GABA_B_ receptors, separately. Our data showed that the blockade of GABA_A_ receptors totally altered vascular response derived from electrical stimulation. These data, suggesting the involvement of GABA_A_ receptor on functional hyperemia, are consistent with recent observations showing that GABA_A_ receptor antagonism reduced CBF response to whisker or basal forebrain stimulation (Kocharyan et al., 2008; Lecrux et al., 2011). Collectively, our findings suggest that GABA_A_ receptor-mediated vascular response is affected by chronic stress.

### Contribution of nNOS-expressing neurons on altered NVC in chronically stressed mice

There are highly diverse populations of GABAergic interneurons in the neocortex (Rudy et al., 2011; Kepecs and Fishell, 2014), and VIP-, SOM-, NOS-, ChAT-, and PV-expressing GABA interneurons are selectively recruited by different afferent inputs (Lecrux and Hamel, 2016). They have been implicated in bidirectional effects dependent on their vasoactive messengers (e.g., vasodilation derived by VIP/NOS and vasoconstriction derived by SOM/NPY) (Cauli et al., 2004; Uhlirova et al., 2016). In this study, we demonstrated that only in the presence of nNOS inhibitors does the vasodilation response of the control group convert to vasoconstriction, thereby mimicking the stressed group. This is consistent with previous studies showing that CBV and CBF responses evoked by photo-stimulation of vesicular GABA transporter was diminished under an NOS blocker (Vazquez et al., 2018), and functional hemodynamic changes elicited by sensory stimulation were significantly reduced under nNOS specific blockers or in nNOS KO mice (Kitaura et al., 2007; Stefanovic et al., 2007). In addition, we confirmed that chronically stressed mice exhibit a reduction in nNOS-expressing neurons via histological approaches matching the results of our previous study reporting that nNOS expression is decreased in chronically stressed rats using Western blotting (Lee et al., 2015). To sum up, our findings strongly support that NOS interneurons have important role in maintaining NVC.

### Limitations and future perspectives

The current study has several limitations that should be addressed in future research. One issue deals with using anesthetics. We used anesthesia to avoid acute stress effects coming from handling and head fixation. Although urethane anesthesia is known to preserve a stable state of NVC (Sceniak and MacIver, 2006; Berwick et al., 2008; Boorman et al., 2015), we note that neurovascular properties in the awake state can be different in some aspects as suggested by other reports (Martin et al., 2013; Gao et al., 2017). Thus, chronic stress effects on sensory-evoked neurovascular responses in the awake state should be further investigated. Another is that our investigation of the chronic stress effects on nNOS-mediated vascular responses were done only in the *ex vivo* situation. Whether the results would be reproduced *in vivo* during sensory stimulation remains to be addressed. To answer this question, comparing the cortical hemodynamics in the unstressed and stressed animals while modulating nNOS activity with nNOS antagonists would be required. This would lead to a better understanding of chronic stress-induced alteration in NO signaling pathways. Moreover, nNOS neurons' activity is highly related to sleep homeostasis (Gerashchenko et al., 2008). Since nNOS-KO mice show altered sleep homeostasis (Morairty et al., 2013), it would be very interesting to study sleep pattern changes in chronically stressed mice.

In conclusion, our results suggest that chronic stress exposure impairs NVC by affecting the expression and function of nNOS-expressing GABAergic interneurons. Since chronic stress is a contributory factor for the progression of stress-related disorder, these findings may suggest an important role of nNOS signaling pathways in preventing or treating stress-related disorders.
